# An Association Study between Hypoxia Inducible Factor-1alpha (HIF-1α) Polymorphisms and Osteonecrosis

**DOI:** 10.1371/journal.pone.0079647

**Published:** 2013-11-18

**Authors:** Georgia Chachami, Alkmini Kalousi, Loukia Papatheodorou, Aggeliki Lyberopoulou, Vasileios Nasikas, Keiji Tanimoto, George Simos, Konstantinos N. Malizos, Eleni Georgatsou

**Affiliations:** 1 Laboratory of Biochemistry, Faculty of Medicine, School of Health Sciences, University of Thessaly, Biopolis, Larissa, Greece; 2 Institute of Biomedical Research and Technology (BIOMED/CERETETH), Larissa, Greece; 3 Department of Orthopaedic Surgery, Faculty of Medicine, School of Health Sciences, University of Thessaly, Biopolis, Larissa, Greece; 4 Department of Translational Cancer Research, Research Institute for Radiation Biology and Medicine, Hiroshima University, Hiroshima, Japan; Saint Louis University, United States of America

## Abstract

Bone hypoxia resulting from impaired blood flow is the final pathway for the development of osteonecrosis (ON). The aim of this study was to evaluate if HIF-1α, the major transcription factor triggered by hypoxia, is genetically implicated in susceptibility to ON. For this we analyzed frequencies of three known HIF-1α polymorphisms: one in exon 2 (C111A) and two in exon 12 (C1772T and G1790A) and their association with ON in a Greek population. Genotype analysis was performed using PCR-RFLP and rare alleles were further confirmed with sequencing. We found that genotype and allele frequency of C1772T and G1790A SNP of *HIF-1α* (SNPs found in our cohort) were not significantly different in ON patients compared to control patients. Furthermore these SNPs could not be associated with the different subgroups of ON. At the protein level we observed that the corresponding mutations (P582S and A588T, respectively) are not significant for protein function since the activity, expression and localization of the mutant proteins is practically indistinguishable from wt in HEK293 and Saos-2 cells. These results suggest that these missense mutations in the *HIF-1α* gene are not important for the risk of developing ON.

## Introduction

Osteonecrosis (ON) is a disabling disorder mainly affecting the hips of young adults in the third and fourth decade of their life. ON has been associated with a variety of risk factors and can be characterized as idiopathic (of no apparent etiology) or secondary from several etiology-associated factors, such as hyperlipidaemia, hypercortisonism, dysbaric phenomena, autoimmune diseases, endotoxic reactions, alcoholism and smoking [1], [2]. Thrombophilia and hypofibrinolysis have also been associated with the pathogenesis of ON which consequently results to bone ischemia, apoptosis of the osteocytes and the bone marrow and final establishment of a bone infarct. [3].

Low oxygen tension, also known as hypoxia, is one of the main pathophysiological characteristics of osteonecrosis [4]. Hypoxia can also occur in many other known diseases like ischemic conditions [5] (myocardial infarct, strokes), cancer [6] or even osteoarthritis [7]. HIF-1 (Hypoxia inducible factor-1) is the major transcriptional regulator triggered in hypoxia in order to promote adaptation to the new environment [8]. HIF-1 binds to regulatory DNA sequences called Hypoxia-Response Elements (HRE) thus controlling the expression of genes involved in cell metabolism, erythropoiesis, angiogenesis, invasion and metastasis. It is a heterodimer that consists of HIF-1α, which is the oxygen regulated subunit, and the constitutively expressed HIF-1β [9]. Under normal oxygen conditions, HIF-1α is continuously produced and destroyed in a process involving hydroxylation of two prolines within the oxygen-dependent degradation domain (ODD). Hydroxylation is mediated by conserved prolyl hydroxylases (PHDs), which act as oxygen sensors. Under hypoxic conditions, hydroxylation is impaired and HIF-1α is stabilized, it translocates to the nucleus where it dimerizes with HIF-1β, thus promoting the transcription of its target genes [10].

The role of HIF-1α in osteonecrosis still remains elusive. It was shown that HIF-1α is significantly increased in the ischemic side of the epiphysial cartilage in the femoral head [11]. HIF-1α was also detected at the reparative zone of collapsed femoral heads of ON patients [12]. Moreover, VEGF (Vascular Endothelial Growth Factor), which is a target of HIF-1α, was also increased in ischemia of femoral head, leading to revascularization of the cartilage thus promoting remodeling after the collapse [11], [12]. The importance of HIF-1α to the cartilage was known before, since it has been reported that HIF-1α is essential not only for cell growth and survival of growth plate chondrocytes in vivo [4], [13] but also for extracellular matrix formation [14]. Latest studies suggest that hypoxia promotes via HIF-1α a metabolic reprogramming in bone marrow–derived angiogenic cells which leads to increased survival in ischemic tissue [15]. For these reasons HIF-1α could be a protein of great significance in the pathogenesis or reparative progress of ON. Furthermore many genetic risk factors (mostly associated with coagulation and fibrinolysis) were already been linked to the pathogenesis of ON [16]. For these reasons we investigated the genetic implication of three well-known and characterized polymorphisms (C111A in exon 2 and C1772T and G1790A in exon 12) of HIF-1α in the appearance of ON.

## Materials and Methods

### Study subjects

The study population consisted of 134 ON patients (88 male, 46 female; mean age, 39.8 years ranging 18–69 years) and 63 controls (47 male, 16 female; mean age, 36.6 years ranging 17–59 years). ON diagnosis were established by evidence of osteonecrosis through magnetic resonance imaging (MRI) in stage 1 of the Association Research Circulation Osseous (ARCO) classification system and plain radiographs in Stages 2, 3, and 4.

According to etiological factors, patients were subgrouped into three groups of ON, group 1: idiopathic (32 cases), group 2: steroid-induced (78 cases), and group 3: other etiologies including alcohol-induced, anemia etc (24 cases). Patients with possible combined causes were excluded. Healthy control subjects were defined as individuals with no significant medical problems, no hip pain and their anteroposterior and frog leg lateral pelvic radiographs did not show any lesions with a sclerotic margin or subchondral collapse consistent with ON.

Idiopathic ON was defined by exclusion of cases with steroid-induced ON, alcohol-induced ON or possible combined cases. Steroid-induced ON was defined by a history of taking prednisolone (1,800 mg) or an equivalent over 4 weeks [17]. Underlying diseases in steroid-induced ON were allergic respiratory or cutaneous diseases (21 cases), systemic lupus erythematosus (20 cases), organ transplantation (13 cases), idiopathic thrombocytopenia purpura (11 cases), nephritic syndrome (8 cases), and others (5 cases). Alcohol-induced ON was diagnosed by the consumption of more than 400 ml of pure ethanol per week or alcohol-induced fatty liver and liver cirrhosis [18].

Clinical profiles of all patients used in this study are summarized in Table 1. There were no significant differences between patients and normal controls, in terms of age, sex, BMI and appearance of diabetes.

**Table 1:** Clinical characteristics of controls and ON patients used in this study.

	Control (N = 63)	Patients (N = 134)	Idiopathic (n = 32)	Steroid-induced (n = 78)	Other etiology (n = 24)	P*
**Age, mean (range)**	36.6 (17–59)	39.8 (18–69)	46.6 (23–69)	37.3 (18–69)	38.5 (18–62)	0.086
**Sex (male/female)**	47/16	88/46	22/10	45/33	19/5	0.25
***Concurrent or past medical history***						
**Hypertension (%)**	0 (0%)	21 (15.7%)	7 (21.8%)	9 (11.5%)	5 (20.8%)	<0.001
**Hyperlipidemia (%)**	0 (0%)	11 (8.2%)	5 (15.6%)	4 (5.1%)	2 (8.3%)	0.018
**Diabetes mellitus (%)**	0 (0%)	5 (3.7%)	2 (6.2%)	2 (2.5%)	1 (4.1%)	0.179
**Renal diseases (%)**	0 (0%)	13 (9.7%)	0 (0%)	13 (16.7%)	0 (0%)	0.01
**SLE (%)**	0 (0%)	20 (14.9)	0 (0%)	20 (25.6%)	0 (0%)	<0.001
**BMI (kg/m2)**	25.8	25.75	26.25	25.82	24.87	0.406

* P values for differences between patients and controls.

The study was approved by the Institutional Review Board (IRB) of the University Hospital of Larissa (Ref. N. 47714, 13/12/2012). Verbal informed consent was obtained from all participants in the study instead of written consent because the data were analyzed anonymously. The method of obtaining verbal consent was approved by the IRB (for minors, consent was obtained from their parents). The procedures followed were in accordance with the ethical standards of the IRB at our institution (University Hospital of Thessaly, Larissa, Greece) on human experimentation and with the Helsinki Declaration of 1975, as revised in 2000.

### DNA extraction and PCR

Genomic DNA was isolated from peripheral blood by using a Nucleospin Blood DNA extraction kit (Macherey-Nagel). The primers used for the exon 12 region were the following: forward 5′-GCT GAA GAC ACA GAA GCA AAG AAC-3′, reverse 5′-GGG TAG GAG ATG GAG ATG CAA TCA-3′. The primers for the C111A region were described in Konac et al [19]. The PCR conditions used were: denaturation at 95°C for 4 min, followed by 30 cycles of denaturation at 95°C for 30 s, annealing and extension for exon 12: 57°C for 2 min and 72°C for 2 min, for exon 2: 60°C for 30sec and 72°C for 30 sec, followed by a final extension step at 72°C for 10 min. All PCR products were further purified using the Nucleospin Gel and PCR clean up kit (Macherey-Nagel).

### RFLP (Restriction Fragment Length Polymorphism) and Sequence analysis

The three polymorphisms were analyzed by digesting O/N at 37°C the PCR products with restriction endonuclease BglII for C111A, HphI for C1772T and AciI (Fermentas, USA) for G1790A. The products were separated by a 2% agarose gel electrophoresis. Samples characterized by RFLP as heterozygous or homozygous for the rare allele were analysed further and verified by sequence analysis using the ABI PRISM BigDye Terminator v3.1 Ready Reaction Cycle Sequencing Kit in a ABI 3100/3130 Sequencer.

### Statistics

Chi-square testing was used to determine the fit between observed genotype frequencies and those expected under Hardy–Weinberg equilibrium (HWE) using an online calculator tool http://www.tufts.edu/~mcourt01/Documents/Court%20lab%20-%20HW%20calculator.xls. Frequencies of discrete variables between ON patients and controls were compared with the Chi-square test (fisher's exact test) and continuous variables were compared by Student's t test or Mann-Whitney test (non parametric variables). Allelic or genotype associations between SNPs in control and ON patients, were compared using 2×2 contingency tables and the 2-sided Fisher exact test. The strength of association was estimated by the odds ratio (OR), and their 95% confidence intervals (CI) were calculated. Statistical significance in luciferase assays was measured using the Student's t-test. A P value of less than 0.05 was considered to be statistically significant (*P<0.05, ***P<0.001). All statistics were calculated through Graph Pad Prism 5 software.

### Cell cultures, plasmids and reporter assays

HEK293 and Saos-2 cells were maintained in Dulbecco's medium supplemented with 10% fetal calf serum plus penicillin (50 IU/ml) and streptomycin (50 mg/ml). pFLAG-CMV2-wild-type HIF-1α and mutant (P582S and A588T) constructs were described in Tanimoto et al [20]. Transient co-transfection of HIF-1α constructs with a reporter gene pGL3–5HRE-VEGF was carried out using the TransPassTM D2 Transfection Reagent (New England Biolabs Inc., Beverly, MA) and according to the manufacturer's instructions.

The transcription activity of HIF-1α constructs was analyzed in a luciferase assay (described in [21]). Localization of HIF-1α transfected constructs in Saos-2 and HEK293 cells was determined using immunofluoresence (described in [21]) using an anti-FLAG antibody (Sigma). Protein expression of FLAG constructs was analyzed in Western blot using a homemade anti-HIF-1α antibody [22] or a monoclonal anti-HIF-1a antibody (BD Biosciences) and anti-tubulin (Milipore, Billerica, MA,USA), as loading control.

## Results

**Figure 1:**
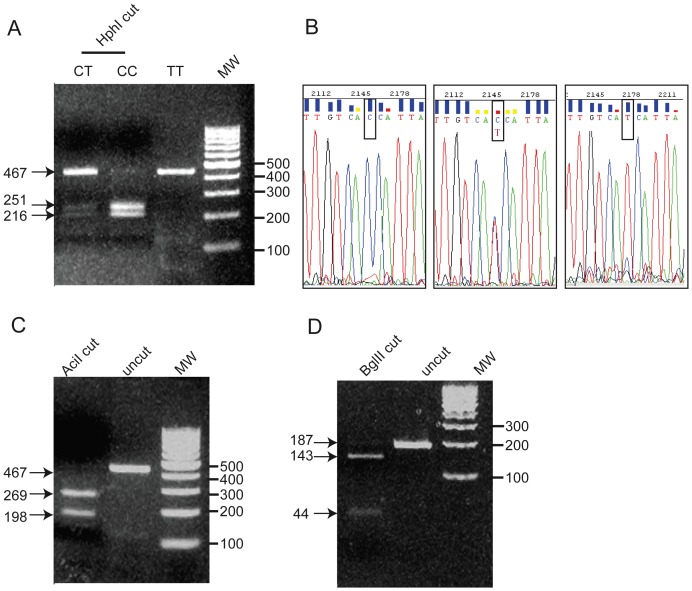
Analysis of HIF-1α polymorhisms using RFLP. (A) Analysis of HIF-1α gene C1772T polymorphism with HphI enzyme, shown on 2% agarose electrophoresis. CT heterozygous genotype yielded three bands 467, 251 and 216 bp; C allele wt yielded two bands (251 and 216 bp). T allele remained uncut and yielded one fragment at 467 bp. Molecular weight standards are shown on the right. (B) Chromatograms of DNA sequence analysis of HIF-1α exon12 fragment showing the corresponding C, CT, T allelic variations at position 1772. (C) Analysis of HIF-1α gene G1790A polymorphism with AciI enzyme, shown on 2% agarose electrophoresis. G allele wt yielded two bands (269 and 198 bp). A allele would remained uncut and yielded one fragment at 467 bp, as represented. Molecular weight standards are shown on the right. (D) Analysis of HIF-1α gene C111A polymorphism with BglII enzyme, shown on 2% agarose electrophoresis. C allele wt yielded two bands (143 and 44 bp). A allele would remained uncut and yielded one fragment at 187 bp. Molecular weight standards are shown on the right.

In order to examine the association of HIF-1α gene polymorphisms with the susceptibility to osteonecrosis (ON) we analyzed the genotype and allele frequencies of three known SNPs located in HIF-1α translated regions (C111A in exon 2, and C1772T and G1790A in exon 12 [23]) between ON patients of different etiologies and control subjects. Analysis of the genotypes was performed on PCR fragments amplified from DNA extracted from peripheral blood using specific restriction endonucleases (Fig 1, A, C, D). To confirm the polymorphisms found, sequencing of the PCR fragments of all subjects carrying the rare allele was performed (Fig 1B for C1772T SNP).

Concerning the C1772T SNP there was no significant deviation from the Hardy-Weinberg equilibrium in control (P = 0.777) and patient group (P = 0.367, Table 2). Out of the 63 controls, 50 cases were type CC, 12 cases were type CT and 1 case was type TT. Concerning patients diagnosed with ON, out of the 134 cases, 101 cases were type CC, 32 cases were type CT and 1 case was type TT. Statistics showed no significant differences in genotype (P = 0.592, OR (95% CI) = 1.257 (0.608 to 2.597)) and allele (P = 0.742, OR (95% CI) = 1.162 (0.600 to 2.254)) frequencies for the C1772T polymorphism, between ON patients and controls (Table 3). In accordance with a previous corresponding study in a Korean population, that similarly did not find association between the C1772T SNP and ON in the whole patient sample, but detected however an association within a subgroup [24], we discriminated subjects in males and females and in subgroups of ON, depending on the etiology of appearance. When we compared control cases versus different ON subgroups (1: idiopathic, 2: steroid induced and 3: other etiologies), we still could not find a significant difference in genotype (P vs idiop.  = 1, OR (95% CI) = 0.887 (0.302 to 2.607), P vs steroid.  = 0.433, OR (95% CI) = 1.417 (0.644 to 3.120), P vs other etiol.  = 0.773, OR (95% CI) = 1.282 (0.436 to 3.881), Table 4) nor in allele frequencies (P vs idiop.  = 0.807, OR (95% CI) = 0.827 (0.302 to 2.267), P vs steroid.  = 0.590, OR (95% CI) = 1.244 (0.605 to 2.560), P vs other etiol.  = 0.604, OR (95% CI) = 1.366 (0.515 to 3.623), Table 4). When we used the male population in our subgroups for the analysis, the difference still remained non significant (data not shown).

**Table 2:** Frequencies of HIF-1α polymorphisms between ON patients and controls.

SNPs	POSITION	GENOTYPE								FREQUENCY*		HWE**	
		control				patients				control	patients	control	patients
**C1772T**	exon 12	CC	CT	TT	N	CC	CT	TT	N	0.111	0.126	0.777	0.367
		50	12	1	63	101	32	1	134				
**G1790A**	exon 12	GG	GA	AA	N	GG	GA	AA	N	0.024	0.011	0.846	<0.0001
		60	3	0	63	132	1	1	134				
**C111A**	exon 2	CC	CA	AA	N	CC	CA	AA	N	0	0	nc	nc
		63	0	0	63	134	0	0	134				

* Frequencies of rare alleles.

** P values of deviation from HWE, in patients and controls.

**Table 3:** HIF-1α SNP genotype and allele frequencies between ON patients and control subjects.

SNPs	n (%) ON patients	n (%) controls	P-value*	OR (95% CI)**
C1772T	n = 134	n = 63		
*Genotypes*				
CC	101 (75.4)	50 (79.4)		
CT	32 (23.9)	12 (19)		
TT	1 (0.7)	1 (1.6)		
CT+TT	33 (24.6)	13 (20.6)	0.5917	1.257 (0.6080 to 2.597)
*Alleles*				
C	234 (87.3)	112 (88.9)		
T	34 (12.7)	14 (11.1)	0.7425	1.162 (0.5996 to 2.254)
**G1790A**				
*Genotypes*				
GG	132 (98.5)	60 (95.2)		
GA	1 (0.75)	3 (4.8)		
AA	1 (0.75)	0 (0)		
GA+AA	2 (1.5)	3 (4.8)	0.3297	0.303 (0.04932 to 1.862)
*Alleles*				
G	265 (98.9)	123 (97.6)		
A	3 (1.1)	3 (2.4)	0.3894	2.154 (0.4285 to 10.83)
**C111A**				
*Genotypes*				
CC	134 (100)	63 (100)		
CA	0 (0)	0 (0)		
AA	0 (0)	0 (0)		
CA+AA	0 (0)	0 (0)	nc	nc
*Alleles*				
C	268 (100)	126 (100)		
A	0 (0)	0 (0)	nc	nc

* Fisher chi-square analysis, P values <0.05 are considered as statistically significant.

** OR, Odds ratio; CI, confidence interval.

Calculations were performed for genotypes: CC vs CT+TT, GG vs GA+AA.

nc: not calculated.

**Table 4:** HIF-1α SNP genotype and allele frequencies between subcategories of ON patients and control subjects.

SNPs	n (%) controls	n (%) ON patients			P-value*			OR (95% CI)**		
		idiopathic	steroid induced	other etiology	vs idiop.	vs steroid	vs other etiology	vs idiop.	vs steroid	vs other etiology
C1772T	n = 63	n = 32	n = 78	n = 24						
*Genotypes*										
CC	50 (79.4)	26 (81.25)	57 (73.0)	18 (75)						
CT	12 (19)	6 (18.75)	21 (27.0)	5 (21)						
TT	1 (1.6)	0 (0)	0 (0)	1 (4)						
CT+TT	13 (20.6)	6 (18.75)	21 (27.3)	6 (25)	1.000	0.433	0.773	0.887 (0.302 to 2.607)	1.417 (0.644 to 3.120)	1.282 (0.436 to 3.881)
*Alleles*										
C	112 (88.9)	58 (90.6)	135 (86.5)	41 (85)						
T	14 (11.1)	6 (9.4)	21 (13.5)	7 (15)	0.807	0.590	0.604	0.827 (0.302 to 2.267)	1.244 (0.605 to 2.560)	1.366 (0.515 to 3.623)

* Fisher chi-square analysis, P values <0.05 are considered as statistically significant.

** OR, Odds ratio; CI, confidence interval.

Calculations were performed for genotypes: CC vs CT+TT.

Regarding the G1790A SNP there was no deviation from the Hardy-Weinberg equilibrium for the control group (P = 0.846) but there was a significant deviation for the patient group, probably due to the low sample number and the rarity of our mutant allele (P<0.0001, Table 2). Out of the 63 controls, 60 cases were type GG, 3 cases were type GA and we found no case of type AA. Concerning total ON patients, out of the 134 cases, 132 cases were type GG, 1 case was type GA and 1 case was type AA. Statistics showed again no significant differences in genotype (P = 0.3297, OR (95% CI) = 0.303 (0.049 to 1.862)) and allele (P = 0.3894, OR (95% CI) = 2.154 (0.428 to 10.830)) frequencies for this polymorphism between ON patients and controls (Table 3). Due to the small number of cases carrying the rare allele, we didn’t further compare controls with subgroups of ON patients.

As for C111A SNP, we did not find the rare A allele in controls nor in any of the subgroups of ON patients. Therefore, no association was attempted between controls and ON patients for this particular SNP (see Table 3).

**Figure 2:**
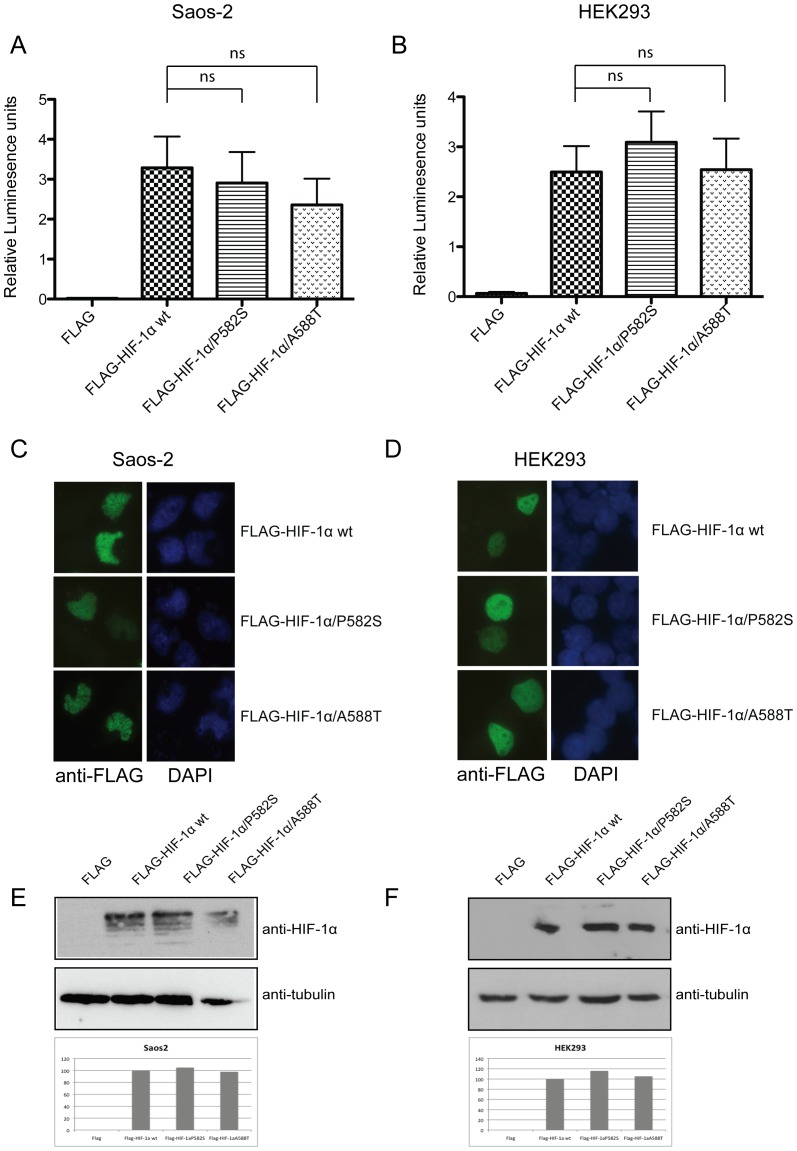
P582S and A588T mutations do not affect HIF-1α transcriptional activity, localization and expression. HIF-1α transcriptional activity was determined 24 h after co-transfection of Saos-2 (A) and HEK293 cells (B) with plasmids expressing the indicated proteins together with the pGL3–5HRE-VEGF reporter plasmid. Values (as relative luminescence units) were determined as a ratio of firefly luciferase activity over Renilla luciferase activity and represent the mean of four different experiments performed in triplicate (+/- S.E.). Statistical differences were assessed using unpaired-t-test. P values>0.05 were considered as statistically non-significant (ns). Saos-2 (C) and HEK293 cells (D) where transfected with plasmids expressing the indicated proteins and their localization was observed after 24 h, with immunofluoresence, using an anti-FLAG antibody. Protein levels of FLAG-HIF-1α wt and mutants were determined in Saos-2 (E) and HEK293 (F) cells after 24-48 h post-transfection, in Western blot using a monoclonal anti-HIF-1α antibody and anti-tubulin as loading control. Densitometric analysis of the bands was performed with the public domain software for image analysis ‘ImageJ’. FLAG-HIF-1α quantities were normalized against corresponding tubulin and expressed as fold increase against wt FLAG-HIF-1α (100%).

Since the SNPs (C1772T and G1790A) that we tested were suggested not to be statistically significant concerning the appearance of ON, we further tried to validate our negative result by using a biological approach. For this we investigated the functional significance of the specific allelic variations in the HIF-1α locus, at the protein level. At first we investigated the activity of the variants using a transcription activity assay with a pGL3–5HRE-VEGF luciferase reporter construct. We co-transfected osteosarcoma Saos-2 and HEK293 cells with the reporter plasmid together with FLAG-HIF-1α wt or with the C1772T mutant (corresponds to FLAG-HIF-1α/P582S) and the G1790A mutant (corresponds to FLAG-HIF-1α/A588T, respectively [20]). Transfection with HIF-1α wt led to a 2.5 to 3fold increase in transcriptional activity both in Saos-2 and in HEK293 cells (Fig. 2, A and B). In Saos-2 cells the expression of both P582S and A588T variants originally led to a small decrease in activity compared to that observed in wt but this was proven not to be statistically significant (Fig. 2A, P_vs P582S_ = 0.7338, P_vs A588T_ = 0.3724). That was also the case for HEK293 cells, where the small differences observed between the activity of the variants were also proven not significant (Fig. 2B, P_vs P582S_ = 0.4652, P_vs A588T_ = 0.9537). Importantly enough, the variations observed between each mutant and the wt molecule showed different traits between the two cell lines tested. This fact implies their lack of significance as was already demonstrated by the statistic analysis. Since we excluded differences in transcriptional activity between HIF-1α variants we next tested if localization or protein expression of HIF-1α is affected by these two mutations. We showed that both HIF-1α P582S and HIF-1α A588T remain nuclear when transfected in Saos-2 and HEK293 cells, exactly like wt (Fig. 2C and D, respectively). Protein levels tested in both Saos-2 and HEK293 cells also remain unchanged between wt and mutants (Fig.2E and F).

In conclusion, in the present study we showed that C1772T and G1790A SNPs found in translated loci of HIF-1α are not associated with the occurrence of osteonecrosis. This could be because these specific polymorphisms seem to have no consequence in the overall activity or protein expression of HIF-1α.

## Discussion

Hypoxia is a main characteristic of several diseases like cancer, ischemic disorders and bone disorders like osteonecrosis and osteoarthritis, where it may also play a pivotal role in their pathogenesis [4], [5], [7], [8]. Osteonecrosis (ON) is a pathological condition that has been attributed to the reduction or loss of vascular supply. This results in the lack of oxygen and consequently to progressive collapse of the bone [3], [25]. HIF-1α is the key transcription factor activated upon lack of oxygen in order to promote the transcription of a great number of genes needed for adaptation to hypoxic conditions [9]. HIF-1α polymorphisms have been extensively studied in order to determine the association they may have in the appearance or progression of hypoxia related diseases. Among many (more than 30) polymorphisms, three are present in exons of HIF-1α (C1772T, G1790A, C111A). Moreover, two of them (C1772T, G1790A) are in the ODD domain of the HIF-1α gene, where they lead to ORF changes from Pro to Ser at codon 582 (C1772T) and from Ala to Thr at codon 588 (G1790A) [23], respectively. It has been shown before that C1772T and G1790A SNPs could be of importance in various types of cancer or specific disorders (like type II diabetes [26], [27] and heart ischemic incidents [28], [29]). However the genetic association or the level of importance they have in the etiology, progression or prognosis of these diseases is controversial among groups and remains subject of ongoing debate. Quite controversial are also the existing reports about the significance of these SNPs in the protein expression or function of HIF-1α. There are reports [20], [30], [31] suggesting that P582S and A588T HIF-1α transcriptional activities are higher than wild type. Yamada et al [26] found that transcriptional activities of these mutants differ significantly from wt HIF-1α only under hypoxia. It was also shown that P582S HIF-1α protein is expressed more compared to the wt [31], [32], maybe due to increased stability under normoxic conditions [31]. Furthermore, there is one study suggesting that the P582S mutation (or in combination with another mutation) may present decreased transcriptional activity compared to wt [29]. None of these studies has however addressed the mechanism underlying the alleles' influence on trigger/progression of the disease studied.

Concerning osteonecrosis, little is really known about HIF-1α SNPs. On the contrary, VEGF SNPs' role has been extensively studied, since VEGF is implicated in neovascularization of the necrotic bone, bone repair and formation [13], [33]. It was shown that SNPs in the promoter region of VEGF are associated with ON of femoral head [34], [35], [36].

In the present study we have investigated the correlation of C1772T, G1790A and C111A HIF-1α polymorphisms, with the appearance of osteonecrosis in a Greek population. We found that the C111A allele was so rare that statistical analysis was not possible. For the other two SNPs (C1772T, G1790A) we could not detect a statistically significant association with the appearance of ON. This is in agreement with the only previous association study about HIF-1α SNPs and ON (but of femoral head) in a Korean population, where the authors have shown no significant association between four SNP genotype and allele frequencies of HIF-1α (including C1772T in exon 12) and ON. Nevertheless in a sub-analysis of the same study, the authors propose that C1772T SNP could be of importance only in the idiopathic ON male group [24]. To check this hypothesis in our own cohort we divided our ON patients into 3 etiological subgroups (idiopathic; steroid induced; of other etiologies) and discriminated between men and women. Our sample number was rather poor in some of our subcategories (especially the idiopathic cases) mostly due to the rarity of such cases in our population. Additionally, our control group was also rather small (but comparable to the different patient subcategories) due to the strict limitations we followed in order to exclude any ON indications and appropriately match with the patients' clinical profile in respect to age, sex, BMI etc (see ‘Study Subjects’ under ‘Materials and Methods’). Despite the low number of patient samples in our subcategories we still did not observe a significant difference or at least a trend showing that these specific polymorphisms of HIF-1α could be genetically implicated with the appearance of any kind of ON.

Our biological approach in two different cell lines showed that *HIF-1α* mutants C1772T and G1790A do not differ from the wt at the protein level since they had no consequence on the localization, expression and activity of HIF-1α protein. To our knowledge this is the first study where the sub-cellular localization of the P582S and A588T mutants has been monitored and one of the very few where their protein levels are compared to wt. These findings concur with/and supplement our genetic results implying that the P582S and A588T mutations might not be genetically important for the appearance of ON because they do not have a profound impact on the overall function of the protein.

To summarize, we found no association of the two known SNPs of HIF-1α with the risk of developing ON of any etiology. We thus believe that these specific SNPs of HIF1α do not have a significant genetic effect on ON diagnosis. Nevertheless a possible implication of other known SNPs of HIF-1a with ON cannot be excluded. Since hypoxia is a key feature of ON, we might expect that downstream effectors of HIF-1α, like VEGF or other angiogenic or metabolic agents could be of value in the diagnosis, progression and therapy of bone diseases like osteonecrosis. However, further biological and functional analysis would be needed to confirm that.

## Acknowledgments

We acknowledge Dr. I. Mylonis for experimental help and support.
